# Epigenetic dynamics and interplay during spermatogenesis and embryogenesis: implications for male fertility and offspring health

**DOI:** 10.18632/oncotarget.17479

**Published:** 2017-04-27

**Authors:** Shao-Qin Ge, Sheng-Li Lin, Zheng-Hui Zhao, Qing-Yuan Sun

**Affiliations:** ^1^ Hebei University Health Science Center, Baoding, China; ^2^ Institute for Reproductive Medicine of Hebei University, Baoding, China; ^3^ Center for Reproductive Medicine of Affiliated Hospital of Hebei University, Baoding, China; ^4^ Hebei Research Institute for Family Planning, Shijiazhuang, China; ^5^ The Center for Reproductive Medicine of Peking University Third Hospital, Beijing, China; ^6^ State Key Laboratory of Stem Cell and Reproductive Biology, Institute of Zoology, Chinese Academy of Sciences, Beijing, China; ^7^ University of Chinese Academy of Sciences, Beijing, China

**Keywords:** epigenetic modifications, intergenerational inheritance, spermatogenesis, embryogenesis, reproductive diseases

## Abstract

Mapping epigenetic modifications and identifying their roles in the regulation of spermatogenesis and embryogenesis are essential for gaining fundamental medical understandings and for clinical applications. More and more evidence has shown that specific epigenetic modifications are established during spermatogenesis, which will be transferred into oocyte *via* fertilisation, and play an important role in the early embryo development. Defects in epigenetic patterns may increase the risk of abnormal spermatogenesis, fertilisation failure, early embryogenesis abnormality and several other complications during pregnancy. This review mainly discusses the relationship between altered epigenetic profiles and reproductive diseases, highlighting how epigenetic defects affect the quality of sperm and embryo.

## INTRODUCTION

Epigenetic modifications such as DNA methylation, histone modifications, noncoding RNAs and protamine code are important regulators in a variety of reproductive processes [1–3]. To form highly specialised mature sperm and facilitate the totipotency of the zygote, these epigenetic modifications must undergo dramatic reprogramming to rearrange chromatin structure, and thus the spermatogenesis and early embryogenesis are particularly vulnerable to epigenetic perturbations [4]. Epigenetic alterations in reproductive processes could result in abnormal expression of target genes and further lead to some reproductive diseases, such as male infertility, early embryo development failure and/or other diseases with underlying epigenetic changes in offspring [5, 6].

Genome-wide profiles of epigenetic modifications have been generated for many different types of cells. Such studies are helpful for understanding the cell type changes during spermatogenesis and embryogenesis, and will provide possible therapeutic targets for the treatment of these reproductive diseases [7, 8]. Likewise, epigenetic biomarkers in sperm have a potential role in predicting and preventing certain diseases in offspring [9]. However, given the diversity and complexity of spermatogenesis and early embryogenesis, it is remaining unclear about the dynamics between distinct epigenetic modifications and links across cellular contexts, and the interplay mechanisms of epigenetic regulation are not well established [10]. This review focuses on the epigenetic profiles and their regulation during the spermatogenesis and embryogenesis processes, providing an overview of the current knowledge on the aetiology of male infertility at the epigenetic level.

### Dynamics of epigenetic modifications and their interplay during spermatogenesis and embryogenesis

**Figure 1:**
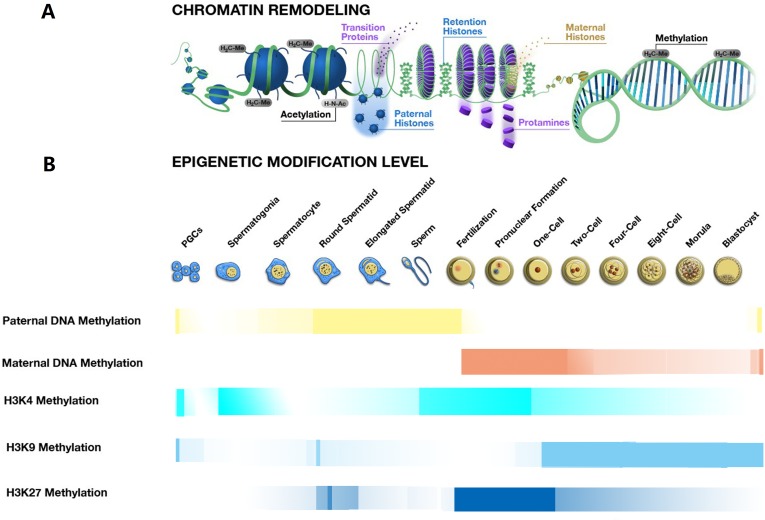
Chromatin remodeling and epigenetic modification changes during spermatogenesis, fertilisation and early embryo development **A**. The process of chromatin remodeling during the spermatogenesis, fertilisation and embryogenesis. The process of chromatin remodeling mainly occurs with the condensation and decondensation of chromatin during spermeiogenesis and after fertilisation. During spermiogenesis from the spermatid to a sperm, the nucleus histones are replaced by the protamine, leading to the chromatin condensation, still with about 15% histone retention in the periphery of the nucleus. After fertilisation, before the pronuclear formation, the protamine-to-histone transition occurs, with the chromatin decondensation, the maternally derived histones replace the sperm protamines. **B**. The transformation of some epigenetic modifications during the spermatogenesis, fertilisation and embryogenesis. The transformations of some epigenetic modifications, including the DNA methylation, H3K4 methylation, H3K9 methylation and H3K27 methylation are essential for sperm production and early embryo development. These epigenetic modifications work cooperatively to regulate phase-specific gene expression that further controls key events in the processes of spermatogenesis, fertilisation and embryogenesis.

Mammalian DNA methylation in cytosine is a critical epigenetic modification that plays crucial roles in transcriptional regulation, chromatin remodelling and genomic imprinting [11–13]. Dynamic erasure and reestablishment of DNA methylation marks catalysed by TET (ten-eleven translocation) dioxygenases and DNA methyltransferases (DNMTs) respectively, are required for the spermatogenesis and embryogenesis [14]. There are two main processes for DNA methylation reprogramming, the first reprogramming process occurs in the onset of spermatogenesis and the other begins at the early stage of embryogenesis (Figure 1). The proper regulation of DNA de novo methylation and demethylation is essential for normal function of the mature sperm and early embryo [1, 15].

In addition, changes of DNA methylation marks may partially rely on pre-existing histone modifications. In the process of de novo DNA methylation, the DNMT3A/B is recruited to H3K9me3 through directly interacting with the HP1 (heterochromatin protein 1) that binds to H3K9me3 *via* its chromodomain, or indirectly interacting with the Suv39h1 and Setdb1 *via* their ADD domain at heterochromatin. At the euchromatin, the DNMT3A/B could combine with euchromatin-associated G9a *via* the MPP8 domain [16]. On the other hand, the H3K4me3 might be involved in blocking the de novo methylation, as the Dnmt3L contains an ADD domain that specifically binds only to the H3K4me0, not to H3K4me3 [16]. Moreover, the H3K4me0 could bind to the ADD domain of the DNMT3A and stimulate the DNMT3A to undergo a significant conformational change from an inhibitory form to an active form [17]. Accordingly, it is speculated that the H3K4me3 might participate in the DNMT3A activity decreased. In the process of DNA demethylation, the H3K9me0 in paternal genome is maintained through binding with the HP1 and occluding the H3K9me1 to prevent further DNA methylation [18]. However in a maternal genome, the DNA demethylation is prevented, since the PGC7 recognises the H3K9me2 and therefore protects the maternal genome from the TET3-mediated hydroxylation. Interestingly, the H3K9me2 also plays a key role in protecting a small subset of the paternal genome from the TET3-dependent cytosine demethylation [19]. In addition, the latest study has shown that the DNA maintenance methylation is largely independent of the H3K9 methylation [20].

As another major epigenetic factor, histone modifications also have critical roles in the establishment of global chromatin environments and orchestration of DNA based biological tasks. There are 16 types of modifications identified to date, which can be considered as biomarkers for both active and inactive regions of the chromatin [21]. In active regions, there are high levels of acetylation and trimethylation at the H3K4, H3K36 and H3K79, while the H3K9, H3K27 and H4K20 methylations are associated with the transcriptional repression [22]. Dynamics of histone modifications are critical for the spermatogenesis and early embryogenesis (Figure 1). Each of these modifications works alone or jointly with others, under the name of ‘histone code’ to promote gene activation or inactivation *via* the disruption of contacts between nucleosomes or the recruitment of non-histone proteins [22]. After fertilisation, the non-canonical H3K4me3 undergoes extensive reprogramming and its level is largely depleted from the early to the late 2-cell-stage embryo. Subsequently, the reestablishment of canonical H3K4me3 occurs rapidly on promoter regions, and many promoters retain this modification till blastocyst stage. Meanwhile, H3K27me3 exhibits a massive loss from the late 2-cell-stage embryo to blastocyst. The distinct features of H3K4me3 and H3K27me3 are essential for zygotic genome activation and the further development [23–25]. As two major mechanisms for the epigenetic regulation, histone modifications and the DNA methylation are expected to regulate the gene expression co-ordinately *via* forming specific chromatin structures. In embryonic stem cells, the H3K27me3 is located in the discrete and punctate regions that are generally devoid of the DNA methylation [16]. From fertilisation to the two-cell stage, the level of H3K27me3 and maternal DNA methylation is high, but the paternal DNA methylation level is decreasing. Accordingly, we could speculate that the H3K27me3 locates preferentially on the paternal genome and excludes from the DNA methylation. Interestingly, promoters that are marked with the H3K27me3 in embryonic stem cells are more likely to gain DNA methylation during the differentiation than those lacking H3K27me3 [16]. The detail mechanism between the H3K27me3 and DNA methylation requires further research.

**Figure 2:**
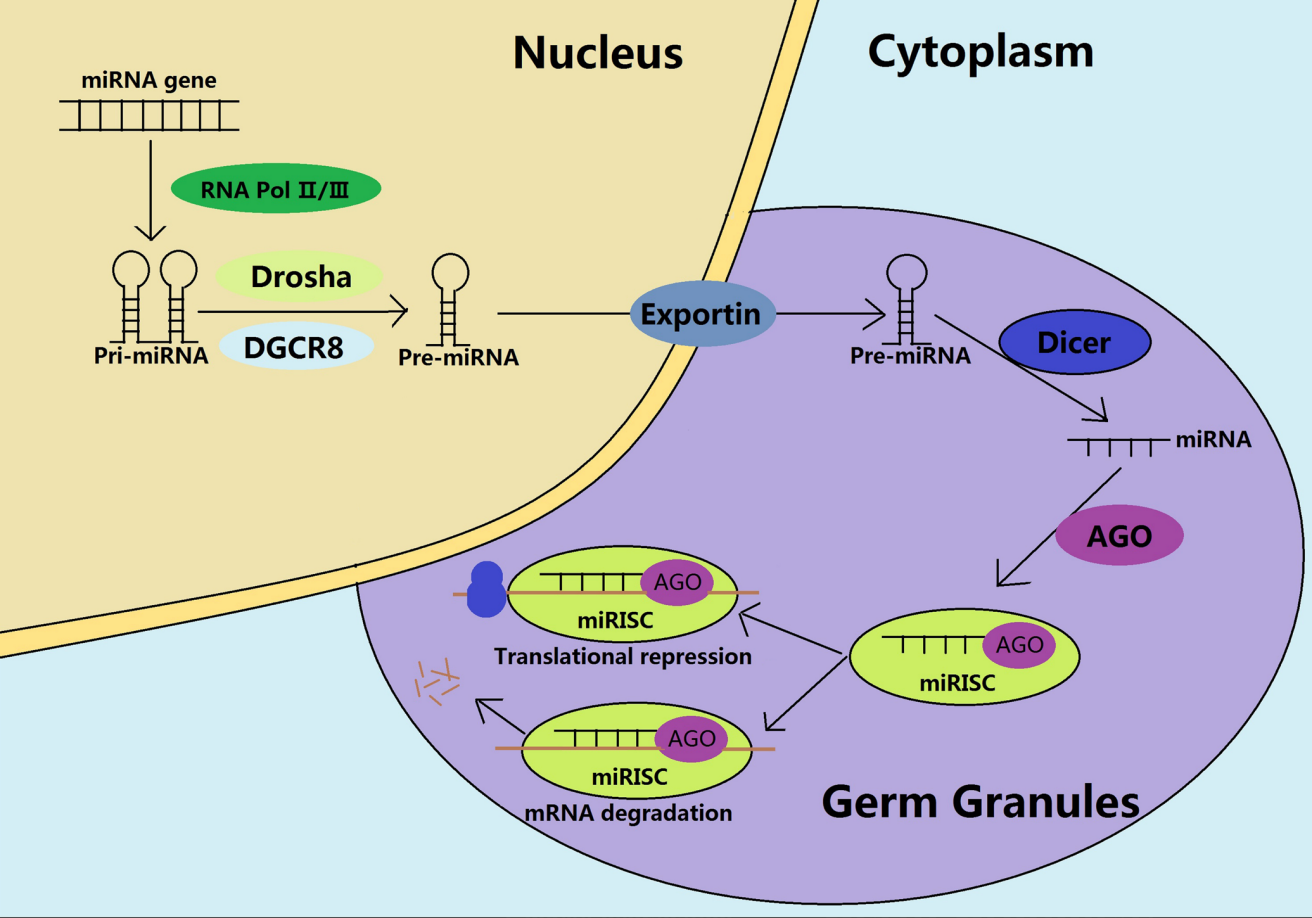
Biogenesis and functions of miRNAs during the spermatogenesis In the nucleus, catalyzed by the RNA Pol II/III, the pri-miRNAs are transcribed from the miRNA genes, and processed into pre-miRNAs through the Drosha and the DGCR8. The pre-miRNAs are subsequently exported to the germ cytoplasmic granules. After maturation by the Dicer, the mature miRNAs unwind and recruit the AGO protein to form a miRNA-induced silencing complex (miRISC), being involved in the mRNA degradation or translational repression, depending on the sequence similarity.

The non-coding RNAs (ncRNAs) represent an important epigenetic modification that exhibits seizes of transcripts and acts as controllers for the gene regulation [26, 27]. They regulate gene expression at the transcriptional and post transcriptional level in different biological contexts and diseases. The miRNAs are 19-25 nucleotides long and involved in an orchestrated stage that specifically controls the gene expression [28]. The main epigenetic effect of miRNAs is to form a miRNA induced silencing complex (miRISC) in the germ granules, facilitating numerous downstream targeted gene silencing by the mRNA degradation or translational repressions (Figure 2). Moreover, miRNAs can also influence the epigenetic phenomena either directly by inhibiting the enzymes involved in the DNA methylation, histone modifications and chromatin remodelling or indirectly by altering the availability of substrates necessary for these enzymatic reactions [27–29]. Interestingly, the same epigenetic modifications regulated by miRNAs can also affect the miRNA expression [27]. For example, the miR-29a and -29b decreases the activities of the DNMT3A and DNMT3B, further impairing the de novo DNA methylation [29]. In addition, the miR-469 could degrade the transcripts of transition protein 2 and protamine 2 in pachytene spermatocytes and round spermatids, which are essential for the successful compaction of the sperm chromatin [30]. Further, some miRNAs are inheritable [31]. For example, the miR-34c is the most abundant in sperm and is also required for the early embryonic cell division through decreasing the expression of B-cell leukaemia/lymphoma 2 (Bcl-2) [32]. It is believed that these inherited miRNAs work with the newly established DNA methylation and histone modifications, to provide an appropriate epigenetic landscape for the embryogenesis.

**Figure 3:**
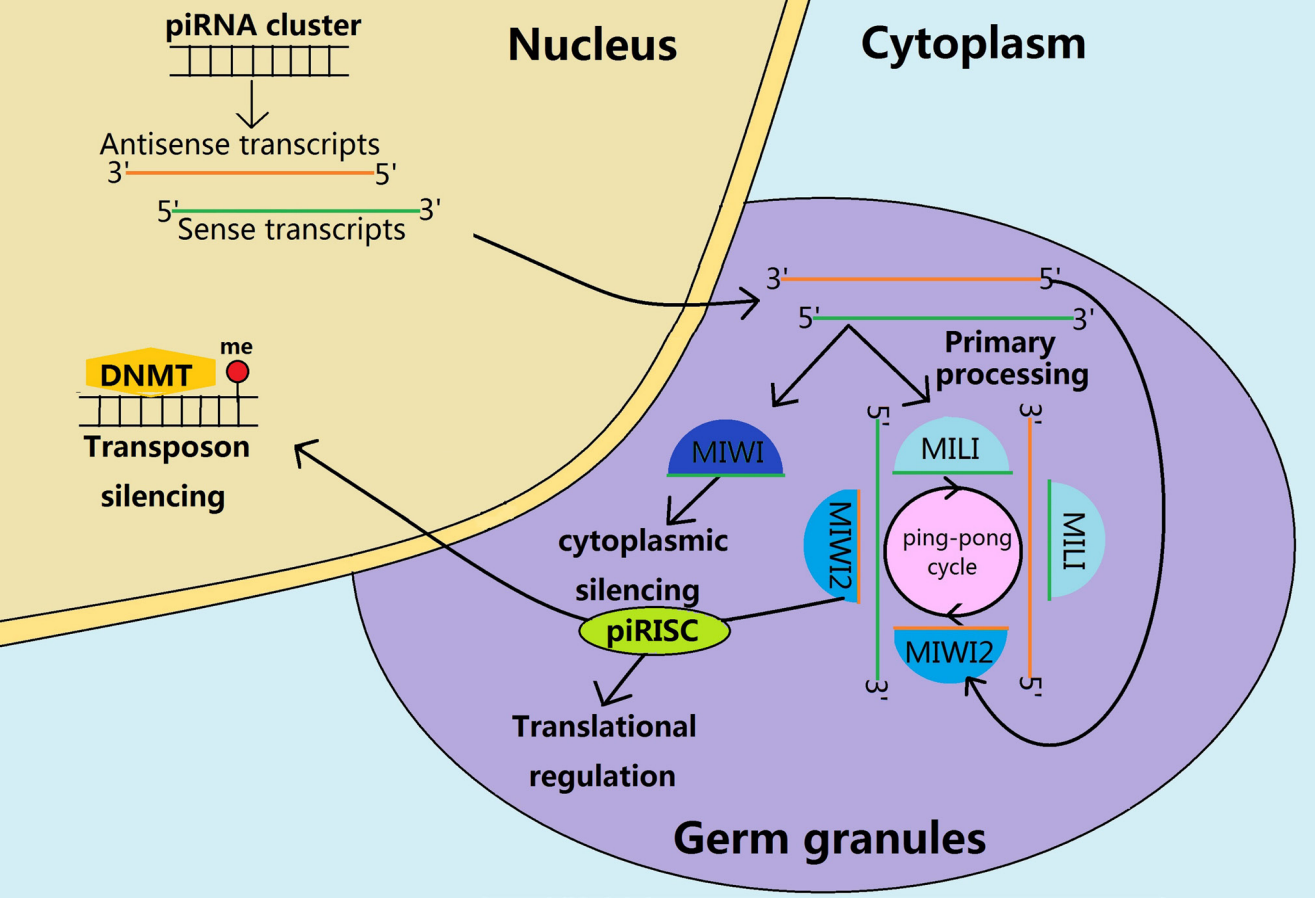
Biosynthesis and functions of piRNAs during the mouse spermatogenesis The sense and antisense piRNA precursors are transcribed from the piRNA clusters in the nucleus and transported to the cytoplasm for further processing in the cytoplasmic germ granules. In a fetal prospermatogonia, the prepachytene piRNAs form piRNA induced silencing complex (piRISC) through the primary processing and the subsequent ping-pong cycle. Subsequently, piRISC participates in translational regulation in cytoplasm and transposon silencing in the nucleus through guiding de novo DNA methylation. After birth, the MIWI2 is no longer expressed but the MIWI appears to have contributed to the cytoplasmatic silencing through primary processing to produce the pachytene piRNAs.

piRNAs are 24-30 nucleotides, a single stranded RNAs that predominantly contribute to the spermatogenesis and embryogenesis in a stage specific manner [27, 33]. Generally, piRNAs are associated with the PIWI proteins and other adjuncts, to form the piRNA induced silencing complex (piRISC) in germ granules (Figure 3). Taking into account the expression patterns of piRNAs and PIWI proteins in male germ cells and early embryos, we believe that piRISC participates in the gene regulation at the transcriptional, post-transcriptional and even translational levels to some extent [34, 35]. The piRISC regulates gene silencing and maintains the genome stability through cleaving of the target mRNAs, or directing the DNA methylation dependent silencing towards target loci in the genome [36].

Transposable elements (TEs) are mobile genetic elements containing a high mutagenic potential when they are expressed unconstrainedly during gametes undergoing the epigenetic reprogramming [37]. Moreover, TEs are regulated by epigenetic modifications that are vulnerable to environmental factors, and such epigenetic information may contribute to environmentinduced intergenerational inheritance [38–40]. In the prospermatogonia, prepachytene piRNAs are necessary for silencing these mobile elements through guiding the de novo DNA methylation at the TEs to guarantee the genome stability [41]. In late spermatocytes and round spermatids, the pachytene piRNAs would be ideal for silencing the retrotransposon sequences through degrading the 3′UTR of retrotransposon mRNAs or recruiting the DNMT3L to the retrotransposon locus [42]. Immediately after fertilisation, both paternal and maternal genomes undergo an epigenetic reprogramming that is accompanied by a spike in retrotransposon expression [38]. piRNAs may protect the embryo from these deleterious elements through inducing epigenetic silencing of retroelements, further preventing the action of various classes of repetitive and transposable elements, including the SINE, LINE, MER and LTR [36, 43].

The histone retention and protamination are two unique processes that specifically exist in the spermiogenesis and early embryogenesis. Recent studies have demonstrated that the histone retention occurred at the spermatogenesis-associated genes, and the regions of retained histones lacked DNA methylation, which might facilitate sperm maturation and fertilisation [44]. Moreover, the retained histones also occurred at the specific developed gene loci and carried epigenetic structural information into early embryo, which might poise genes for activation at specific sites required for the normal embryogenesis [45]. In addition, histone modifications are involved in the retained regions. The H3K4me2 becomes enriched at promoters of developmental genes and is involved in the spermatogenesis and cellular homeostasis, and the H3K4me3 appears at a subset of promoters of the highly expressed developmental genes, paternally expressed as the imprinted loci and the certain noncoding RNA promoters [46, 47]. Moreover, H3K4 methylation and H3K27me3 often co-occur in the histone retained regions, which is a mark involved in polycomb-mediated gene repression without promoting DNA methylation [48]. In addition, the histone variants are also abundant in the retained nucleosomes. Trimethylated histones H3.1 and H3.2 at K27 and histone H3.3 at K4 are enriched in these regions [49].

During spermiogenesis, the protamination plays important roles in the proper condensation of the sperm chromatin [45]. This process directs the histone to protamine transition and forms a structure of nucleosomes within the protamine toroids (Figure 1) that effectively protect the sperm genome against the DNA damage [50]. Recently, the histone variant TH2B was reported to participate in the protamination process. The TH2B could replace the H2B during the spermiogenesis, thereby changing the chromatin forms from histones to protamines [50]. Interestingly, the TH2B could also be involved in the chromatin destabilisation during the early stage of an embryonic development, facilitating the genome plasticity [45]. In addition, the protamine code also contributed to the transcriptional regulation by modulating interactions between protamines and the DNA sequences [45]. During the spermiogenesis process, acetylation and methylation can co-occur on a given protamine, whilst the acetylation and phosphorylation appear to be exclusive to each other [45]. When incorporated into chromatin, the protamines were immediately phosphorylated, which is essential for binding the protein to the DNA. However, once bound to the DNA, the protamines were dephosphorylated, for the proper condensation of chromatin [51]. Mukherjee *et al*. showed that the acetylation of residues *P1 S42* and *P2 S55* could be modified by post-translational modifications, which likely prevented their phosphorylation, providing a possible mechanism for the protamine dephosphorylation [52]. On the other hand, the sperm nucleus remodels extensively in early embryos through replacing the protamines with acetylated histones derived from the maternal genome, leading to the decondensation of the paternal chromatin (Figure 1). This process is the key for generating a transcriptionally competent DNA that helps to facilitate the needs of a zygote [1]. It will be interesting to investigate whether the protamine code serve any function in early embryonic development [46].

## ALTERATION OF EPIGENETIC MODIFICATIONS AND CORRESPONDING DISEASES

Dynamic activation and inactivation of gene expression are essential for driving the differentiation of male germ cells and for the development of early embryos. Identifying the mechanisms of epigenetic regulation is necessary to understand how these fascinating systems contribute to the spermatogenesis and embryogenesis, and how these processes are disrupted in reproductive diseases [10]. The regulation of gene expression is mediated by many epigenetic factors, and abnormalities of these epigenetic profiles may lead to reproductive diseases.

### Abnormal sperm DNA methylation in male infertility and embryogenesis defects

The DNA methylation patterns acquired during spermatogenesis are necessary for a proper sperm production, the fertility and embryo development, and their alterations are closely related to male reproductive health, which can also affect the development of the embryo (Table 1) [6, 53]. Assisted reproductive techniques (ART), especially introcytoplasmic sperm injection, can be applied to treat male infertility by collecting morphology qualified sperm, but this may result in high epigenetic risk for offspring diseases mainly owing to the use of sperm with imprinting disorders. For example, the hypermethylation of the *MEST*-imprinted gene results in the idiopathic infertility for men with a normal sperm morphology below 5% and a progressive sperm motility below 40%, which at least is partly contributed to the Silver-Russell Syndrome (SRS) [54]. There are two differentially methylated regions (DMRs), the *H19* DMR and *KvDMR1*, located on the chromosome 11p15. The hypermethylation of the *H19* DMR and/or hypomethylation of the *KvDMR1* may result in the Beckwith-Wiedemann syndrome (BWS) [55]. Conversely, the hypomethylation of the paternal *H19* DMR may lead to the SRS [54]. Therefore, high epigenetic quality sperm preparation may be a potential way to reduce the risk of epigenetic diseases, which emphasizes the importance of evaluation on the sperm quality in in both genetic and epigenetic levels. On the other hand, some diseases also result from the aberration of DNA methylation in some specific genes. Ferreira *et al*. reported that the specific hypermethylation events in the CpG islands of genes associated with the piRNAs could lead to their transcriptional suppression, which is considered to be a cause of the testicular cancer [56].

**Table 1:** The abnormal DNA methylation and related diseases

Stage	Disease	Aetiology	Species	Reference
Embryogenesis Spermatogenesis	Non-obstructive azoospermia	Hypermethylation at the promoter region of a *MTHFR* can down-regulate the MTHFR expression and, as a consequence, reduce its enzymatic activity	Human	Khazamipour*et al*.[57]
	Azoospermia	Disruption of Dnmt3L in testes results in progressive loss of spermatogonia and further causes complete azoospermia	mouse	Bourc’his*et al*.[68]
	A greatly reduced number of spermatocytes and methylation loss of paternally imprinted genes	Knockout the *Dnmt3a*	mouse	Kaneda *et al*.[64] La Salle *et al*.[65]
	Idiopathic infertility	Hypermethylation of the *MTHFR* genes or the *MEST* imprinted gene	Human	Wu *et al*.[58]Eroglu and Layman[54] Karaca *et al*.[59]
	Idiopathic asthenospermia Testicular germ-cell tumour	hypermethylation of VDAC2 promoter region might reduce sperm motility, ultimately result in idiopathic asthenospermia Hypermethylation of regulatory regions of promoters, associated with the silencing tumour suppressor genes	Human Human	Xu *et al*.[60] Dada *et al*.[61]
	Testicular cancer	Specific hypermethylation events in the CpG islands of genes associated with piRNAs, which leads to their transcriptional inactivation	Human	Ferreira *et al*.[56]
	Retarded gestational growth and failure to result in viable offspring	*Dnmt1* knockout or loss of function mutations in mice decreases the methylation marks globally in embryos, which leads to the expression of biallelic imprinted genes and retrotransposons, and halts the chromosome inactivation	Mouse	Jenkins and Carrell[4]
	Embryonal carcinoma	High expression of DNMT3A, 3B and 3L	Human	Almstrup *et al*.[67]
	Fragile X syndrome	Expansion and methylation of the CGG repeat in FMR15′UTR, promoter for the methylation	Human	Verdyck *et al*.[62] Zhou *et al*.[63]
Birth	Beckwith–Wiedemann syndrome	Hypermethylation of the *H19*-DMR and/or hypomethylation of the *KvDMR1*	Human	Cooper *et al*.[55]
	Silver–Russell Syndrome	Hypomethylation on the paternal allele of the *H19*-DMR or hypermethylated at the *MEST* imprinted gene	Human	Eroglu and Layman[54]

The methylation state of promoter in the genes involved in the spermatogenesis and early embryogenesis is a recent topic of interest. The hypermethylation at the promoter region of the *MTHFR* has shown to down regulate the *MTHFR* expression, and as a result its enzymatic activity is reduced, leading to non-obstructive azoospermia (NOA) and potentially idiopathic infertility [57–59]. Likewise, hypermethylation of VDAC2 promoter region might reduce sperm motility, ultimately result in idiopathic asthenospermia [60]. In the regions of regulatory promoters, which are associated with the silencing of tumour suppressor genes, the hypermethylation can lead to testicular germ cell tumours [61]. Recently, Verdyck *et al*. have confirmed that the expansion of a CGG repeat tract and retained DNA methylation in the *FMR1* 5′UTR promoter resulted in the Fragile X syndrome [62, 63].

In addition, the disruption of DNMTs also resulted in the aberrant DNA methylation profiles clearly, and is associated with some diseases. For an instance, knockout the *Dnmt3a* could greatly reduce the number of spermatocytes and resulted in the methylation loss of paternally imprinted genes [64, 65]. Further, the *Dnmt3a* and *Dnmt3b* knockout experiments have shown that the loss of the DNMT3B could lead to the loss of methylation at a single, specific locus that is absent in the *Dnmt3a* conditional knockouts, indicating there was much redundancy for the DNMT3B function [66]. In contrast, a high expression of the DNMT3A and 3B, as well as their homologue 3L, was closely related to the embryonic carcinoma [67]. The disruption of the Dnmt3L in testes resulted in a progressive loss of the spermatogonia, and further caused a complete azoospermia [68]. The *Dnmt1* knockout or loss of function mutations in mice may reduce the global methylation marks in the embryo, and the expression of the biallelic imprinted genes and retrotransposons were initiated, subsequently embryos exhibited a retarded gestational growth and did not result in a viable offspring [4].

### Abnormal histone modifications associated with male infertility and embryogenesis defects

Histone modifications are shown to have a significant effect on the epigenetic regulation. Specific enzymes are necessary to orchestrate these modifications in sperm and embryos. As a result, the disruption of methylation or acetylation status of histones led to a various degrees of fertility and embryonic loss (Table 2). In addition, the apoptosis, sterility, failure of chromatin condensation, and the peri-implantation lethality are also linked with the perturbation of specific enzymes on the histones [69].

**Table 2:** Abnormal histone modifications and associated diseases

Stage	Disease	Aetiology	Species	Reference
Spermatogenesis	Decrease of spermatocytes	The reduction of histone H3K4 methyltransferase MII2 activity	Mouse	Glaser *et al*.[69]
	Sperm apoptosis and sterility	The loss of LSD1/KDM1	Human	Shi *et al*.[70]
	Impaired post meiotic chromatin condensation	Deficiency of the JHMD2A can down regulate the expression of two genes, the P1 and TNP1, leading to the condensation and proper packaging of the chromatin failure in sperms	Mouse	Najafipour *et al*.[71]
	Inhibit the process of spermatogenesis	Altered dimethylation states of the H3K9	Mouse	Xiong *et al*.[74]
	Nucleosome removal abnormality	Deficiency of the RNF8, a ubiquitin ligase, could lead to the abnormal H4K16 acetylation that significantly suppresses the histone removal and results in the incorporation of the transition protein	Mouse	Lu *et al*.[72]
	Partial failure in chromatin condensation, abnormal sperm head morphology, immotility of epididymal sperm, and male infertility	Knockout *Chd5*, a gene encoding chromatin-remodeling nuclear protein, decreases the H4 hyperacetylation in elongating spermatids	Mouse	Zhuang *et al*.[73]
Embryogenesis	Insufficient sperm chromatin compaction that persists in the zygote	Aberrant acetylation of the H4K12 in promoters of the development of important genes	Human	Paradowska *et al*.[75]
	Less developmentally competent embryos	abnormal expression of BRG1 and KDM1A around the period of embryonic genome activation could alter the H3K4 methylation	Porcine	Glanzner*et al*.[76]
	Peri-implantation lethality	Absence of the ERG-associated protein with the SET domain, a histone methyltransferase that specifically trimethylates the H3K9 residue	Mouse	Dodge *et al*.[77]
Birth	Rubinstein-Taybi syndrome	Acetylation of histones alters the folding of the chromatin nucleoprotein complex	Human	Ausio *et al*.[78]

In the mitosis during spermatogenesis, the reduction of the MLL2 activity, a histone methyltransferase that is specific to the H3K4, could significantly decrease the number of spermatocytes through an apoptotic process [69]. During the meiosis, the loss of the LSD1/KDM1, which is histone demethylases specific to H3K4, could result in the apoptosis of sperm, leading to male sterility [70]. During the spermiogenesis, deficiency of the JHDM2A in mice, a histone demethylase specific to the H3K9, could down regulate the gene expression of the transition protein 1 and protamine 1 [71]. Moreover, deficiency of the RNF8, a ubiquitin ligase, could lead to an abnormal H4K16 acetylation that significantly suppresses the histone removal, and resulted in the transition protein incorporation, leading to the nucleosome removal abnormality [72]. Similarly, knockout the *Chd5*, a gene encoding chromatin-remodeling nuclear protein, would decrease the H4 hyperacetylation in elongating spermatids and further gave rise to a partial failure of the chromatin condensation, abnormal sperm head morphology, immotility of epididymal sperm, and male infertility [73]. In addition, altering the states of the H3K9me2 would inhibit the process of spermatogenesis [74]. All of these abnormal histone modifications have been confirmed to be causes of the defective chromatin condensation and infertility.

During the early stages of an embryogenesis, the aberrant acetylation of H4K12 promoters can reduce the sperm chromatin relaxation, resulting in the zygotic arrest [75]. In addition, decreased expressions of BRG1 and KDM1A around the period of embryonic genome activation could alter the H3K4 methylation and further result in less developmentally competent embryos [76]. Likewise, the absence of ERG associated with the protein, a histone methyltransferase specifically trimethylating the H3K9 residue, have caused the peri-implantation lethality [77]. For example, folding the chromatin nucleoprotein complex altered by the histone acetylation has been considered as a cause for the Rubinstein-Taybi syndrome [78].

### Abnormal noncoding RNAs associated with male infertility

The spermatogenic process is a complex differentiation procedure that is prone to errors [79]. The expression of miRNAs and piRNAs during the spermatogenesis and embryogenesis is significant for the proper production and development of sperm and embryo, respectively. Their altered profiles in the spermatogenesis could result in the male infertility, and may be related to the testicular cancer (Table 3).

**Table 3:** Abnormal non-coding RNAs and male infertility

Stage	Disease	Aetiology	Species	Reference
Spermatogenesis	Reduction of the spermatogonial proliferation and testicular atrophy	Depletion of the E2F1 that can modulate the miR449a/b positively	Mouse	Hoja *et al*.[81]
	Apoptosis of spermatocytes	Over-expression of the E2F1	Human	Marcet *et al*.[80]
	Chromatoid body fragmentation and severe DNA damage	Disruption of the MOV10L1 or the mutation of *MOV10L1* causes a complete loss of piRNAs in pachytene spermatocytes and round spermatids	Mouse	Zheng and Wang[84]
	Spermiogenic arrest	Knockout of the *MIWI* gene	Mouse	Deng and Lin[89]
	Meiotic arrest	Knockout of the *MILI* and *MIWI2* gene	Mouse	Kuramochi-Miyagawa *et al*.[90] Carmell *et al*. [91]
	Male infertility and testicular germ cell tumor	Abnormal testicular miR-383 expression, which can inhibit the expression of a tumor suppressor, interferon regulatory factor-1 (IRF1); the accumulation of NLC1-C in the nucleus of spermatogonia and primary spermatocytes represses both miR-320a and miR-383 transcription	Human /Mouse	Lian *et al*.[86] *Lü et al.* [96]
	Azoospermia	The levels of miR-34c-5p, miR-122, miR-146b-5p, miR-181a, miR-374b, miR-509 -5p, and miR-513a-5p were significantly lower	Human	Wang *et al*.[87]
	Asthenozoospermia	The levels of miR-34c-5p, miR-122, miR-146b-5p, miR-181a, miR-374b, miR-509 -5p, and miR-513a-5p were higher	Human	Wang *et al*.[87]
	Asthenozoospermia or oligoasthenozoospermia	miR-122 were down-regulated in the asthenozoospermia, which is synthesized only in round spermatids, participating in the post-transcriptional down-regulation of the TNP2 through targeting the 3′ untranslated region of the TNP2 mRNA; a decreased expression of HOTAIR, one of lncRNAs, may reduce H4 acetylation in Nrf2 promoter and Nrf2 expression	Human	Abu-Halima *et al*.[85] Zhang *et al*.[95]
	Oligoasthenoteratozoospermia	Loss of miR-34bc/449	Mouse	Comazzetto *et al*.[82]
	Reduced testis size and sperm count, and complete male infertility	Germ cell-specific deletion of the *Dicer1*	Mouse	Romero *et al*.[83]
	Infertility with a complete absence of sperm and testis degeneration	Ablation of Dicer in Sertoli cells	Mouse	Papaioannou *et al*.[88]
Embryogenesis	Sexually dimorphic, partial perinatal lethality, growth retardation, and infertility	Simultaneous inactivation of the *miR-34b/c* and *miR-449*	Mouse	Wu *et al*.[92]

The expression of miR449a/b is positively modulated by the E2F transcription factor 1 (E2F1). Over expression of the miR449a/b would increase the apoptosis of spermatocytes, and reduce the depletion of E2F1 for the spermatogonial proliferation, further resulting in the testicular atrophy [80, 81]. In contrast, the loss of miR449 may lead to oligoasthenoteratozoospermia [82]. On the other hand, the germ cell-specific deletion of *Dicer1* could reduce the testis size, sperm count, and complete male infertility [83]. During the pachytene spermatocyte stage, the disruption of MOV10L1, an RNA helicase, may induce a complete loss of the piRNAs, resulting in the chromatoid body fragmentation [84]. Likewise, the mutation of *Mov10l1* in round spermatids may also stimulate the deficiency of the piRNAs, resulting in severe DNA damages [84]. Similarly, the miR-122 synthesis in round spermatids participates in the process of post transcriptional down regulation of the transition protein 2, through targeting the 3′ untranslated region of the mRNA. The down regulation of miR-122 is a feature of the asthenozoospermia or the oligoasthenozoospermia [85]. In the later stages of spermatogenesis, a decreased expression of the testicular miR-383 could inhibit the tumour suppressor, an interferon regulatory factor-1 (IRF1), which may provoke the male infertility and testicular germ cell tumour [86]. In addition, there are some diseases involving several types of miRNA. For example, levels of the miR-34c-5p, miR-122, miR-146b-5p, miR-181a, miR-374b, miR-509-5p, and miR-513a-5p are significantly lower in patients with the azoospermia and higher in patients with the asthenozoospermia [87]. There are also some proteins that are required for the maturation of ncRNAs, while abnormalities in such protein expression may lead to some diseases. For instance, the ablation of *Dicer* in the Sertoli cells is associated with infertility due to the complete absence of sperm and testis degenerations [88]. The knockout of the *Miwi* gene is shown to result in the spermiogenic arrest [89]. Similarly, knockout of the *Mili* and *Miwi2* could lead to the meiotic arrest [90, 91].

In addition, the sperm miRNAs can be transmitted to embryos following the fertilisation, and the altered profiles of miRNAs in sperm may result in an abnormal embryonic development. For example, the inhibition of the miR-34c can suppress the DNA synthesis and arrest the embryo at the first cleavage division [32]. Simultaneous inactivation of the *miR-34b/c* and *miR-449* would cause sexually dimorphic, partial perinatal lethality, growth retardation, and infertility [92]. Apart from the sperm miRNAs, the seminal miRNAs that are mainly derived from the testis and epididymis are also believed to be useful noninvasive biomarkers for the identification of the impaired sperm production and maturation [93]. For example, in the seminal plasma of patients with non-obstructive azoospermia the levels of the three miRNAs - miR-141, miR-429 and miR-7-1-3p - were found to be increased, compared with the fertile controls [94].

It is apparent that the alterations of miRNAs and piRNAs play key roles in the reproductive diseases, and the lncRNAs are a new addition to the ncRNAs family. LncRNAs transcription and processing are complicated, whilst the majority of them are localized in the nucleus, suggesting that they may be involved in the regulation of chromatin. For example, a decreased expression of HOTAIR may reduce H4 acetylation in *Nrf2* promoter and *Nrf2* expression, which is closely associated with asthenozoospermia and oligoasthenozoospermia [95]. Moreover, the accumulation of NLC1-C in the nucleus of spermatogonia and primary spermatocytes represses both miR-320a and miR-383 transcription by binding to Nucleolin, resulting in hyperactive proliferation of germ cells and further leading to male infertility [96]. These lncRNAs in the genome have numerous intrinsic messages and future research efforts should identify and characterize the lncRNAs that are actively involved in the events of spermatogenesis and embryogenesis in detail.

### Abnormal proportion of protamines associated with reduced male fertility

Protamines are critical for the proper chromatin packing. The variations in sperm protamine expression and ratio have been shown to be associated with the precocious chromatin condensation, the increase in the DNA fragmentation, the lower sperm counts and reduced fertilising capacity, which lead to the embryonic arrest (Table 4) [97].

**Table 4:** Reduced male fertility with an abnormal proportion of protamines

Stage	Disease	Aetiology	Species	Reference
Spermatogenesis	Precocious chromatin condensation, transcription arrest, and spermatogenic failure	Deregulation of protamines	Mouse	Cho *et al*.[5]
	Sperm DNA fragmentation	Abnormally high or low P1/P2 ratios	Human	Simon *et al*.[98, 99, 100]
	Lowered sperm counts	Haploinsufficiency of protamines	Mouse	Castillo *et al*.[100]
	Male infertility	The depletion of P2 leads not only to impaired histone to protamine exchange and disturbed DNA-hypercondensation, but also to severe membrane defects resulting in immotility	Mouse	Schneider *et al*.[101]
	Asthenozoospermia	lower levels of P1 and P2 transcripts or P1/P2 ratio	Human	Kempisty *et al*.[99]
Embryogenesis	Lower pregnancy rates	Abnormal protamine replacement	Mouse	Cho *et al*.[5]
	Embryo lethality	Low P2 concentrations	Mouse	Cho *et al*.[5]

The protamine 1(P1) and protamine 2 (P2) are usually expressed in nearly equal quantities [98]. Kempisty *et al*. have shown that lower levels of the P1 and P2 transcripts or the P1/P2 ratio exist in the ejaculated sperm of asthenozoospermic men, and believed that they were associated with the abnormal retention of the protamine mRNA, which is known to increase the DNA fragmentation level [53, 99]. Simon *et al*. have shown that the abnormally high or low P1/P2 ratios may result in an increased sperm DNA fragmentation [97]. Meanwhile, the down regulation or haploinsufficiency of protamines can induce the precocious chromatin condensation, transcription arrest, spermatogenic failure and lowered sperm counts [100]. Currently, as it is easy to measure, the P1/P2 ratio is widely used as a marker of abnormal spermiogenesis [100]. In addition, reports from many laboratories have shown that a change in the P1/P2 ratio is not only associated with the altered sperm quality, but also with the decreased embryo quality and the IVF outcomes, when compared with infertile patients with a normal P1/P2 ratio. de Mateo *et al*. have found that an abnormal protamine replacement can reduce the pregnancy rate [98].

In addition, the histone to protamine ratio can be used directly to detect aberrations in the histone retention, which has connections with the male infertility. The depletion of P2 leads not only to impaired histone to protamine exchange and disturbed DNA-hypercondensation, but also to severe membrane defects resulting in immotility [101]. In addition, the DNA damage in a murine sperm was associated with the lower P2 concentration and the embryonic arrest was observed when such sperm was used to fertilise an oocyte through an intracytoplasmic sperm injection [5]. These results indicate that protamines may have a greater role in the spermatogenesis and in the early embryo development than previously have believed [1].

## CONCLUSION AND PERSPECTIVES

It is apparent that the dynamics of epigenetic modifications and their regulatory networks are essential for the normal spermatogenesis and embryogenesis processes. The perturbations in any such modifications are likely to cause some degrees of infertility, and the altered epigenetic marks could be inherited and further result in phenotypic defects in the offspring [102]. For instance, a high-fat or low-protein diet in male mice can alter the metabolic gene expression in the offspring, which is mediated by the small noncoding RNAs derived from the transition RNAs [103, 104]. Moreover, it seems more obvious that various altered epigenetic modifications associated with reproductive diseases are all linked together. Clearly, abnormal DNA methylation are in fact associated with altered histone modifications, dysregulation of ncRNAs as well as dysfunctions of protamination, and all of them contribute to male infertility in some degree. Therefore, the research on epigenetic alterations in sperm and embryos is essential for us to understand the pathogenesis of reproductive diseases and develop the novel approaches and applications in the treatment of male infertility and offspring diseases. However, an association between the epigenetic landscape of a given regulatory element, male infertility and embryonic defects is still evolving, and numerous clinical effects of abnormal epigenetic modifications remain to be fully investigated. It is necessary to elucidate the interplay mechanisms for epigenetic modifications, and determine the best timeframe to reverse the deviant epigenetic marks.

Satisfyingly, the epigenetic editing system has represented a powerful toolbox that can be used to drastically modify the epigenetic landscape of a specific regulatory element, and this useful technique may be readily adopted in gene and cell therapies [105, 106]. Meanwhile, identifying specific enzymes for the modulation of abnormal epigenetic marks is also essential for the development of epigenetic drugs that can be used for treatments at the body level. We believe that a highly efficient and specific epigenetic therapy will be an important milestone for the development of precision medicines.
